# Textured surface stereoscopy

**DOI:** 10.1177/20416695251349685

**Published:** 2025-07-25

**Authors:** Nicholas J Wade

**Affiliations:** University of Dundee, UK

**Keywords:** random-texture stereograms, anaglyphs, enclosed and extended depth, graphics, photography, photographics

## Abstract

Julesz constructed stereograms in which surfaces in depth could be seen with two eyes but not with either eye alone. He noted that such enclosed surfaces in depth never occur in natural scenes. In contrast, extended stereoscopic surfaces are a natural feature of binocular vision. Examples of constructed textured surface stereograms are presented as anaglyphs. They satisfy the criterion of revealing depth seen with two eyes which is concealed from each eye alone. A wide range of carrier patterns can be employed to construct complex stereoscopic surfaces. Stereoscopic inclusions can be embedded within modulated surface depths in the same anaglyphs, and conventional stereoscopic images (photographs) can be incorporated within constructed stereograms. Textured surface stereograms offer the possibility of extending the artistic expression of stereoscopy.

## How to cite this article

Wade, N.J., (2025). Textured surface stereoscopy. *i-Perception, 16(4)*, 1–22. https://doi.org/10.1177/20416695251349685 

## Introduction

Investigations of stereoscopic depth perception were transformed by Julesz (1960, 1971) with computer-generated random-dot patterns. An enclosed surface in depth (like a square) was only visible when two seemingly flat arrays of small black and white squares were combined stereoscopically. Julesz noted that “Such visual displays ordinarily never occur in real-life situations” (1960, p. 1126). It has been argued that a precursor of a random-dot stereogram (RDS) can be found in the article describing the first stereoscope (Wheatstone, 1838): a row of five dots was presented to each eye but those in one eye had larger separations than in the other (see Wade, 2025). If the horizontal rows had been extended vertically then a surface slanting in depth would be visible stereoscopically. However, the distinguishing feature of the depth surfaces seen by these pioneers of perception is that they were unbounded for Wheatstone but within defined regions of the stereograms for Julesz. This article is concerned primarily with extended surfaces in stereoscopic depth using textured patterns similar to RDSs. Unlike the enclosed surfaces introduced by Julesz, extended stereoscopic surfaces constantly “occur in real-life situations”. Unbounded surfaces are a fundamental feature of conventional stereoscopic photographs but not of constructed stereograms. That is, in the latter, the monocular patterns (half-images) are made without reference to external objects; stereoscopic depth is introduced by spatial displacements of a region in one half-image but not in the other. The spatial structures of the half-images are referred to as carrier patterns, the most popular of which are computer-generated random-dot textures. Julesz (1960) described RDSs as “patterns devoid of all cues except binocular parallax, by using artificially created stereo images” (p. 1126). The same applies to the textured surface stereograms shown in this article. While the majority of stereoscopic investigations have involved enclosed surfaces, there are also studies of sloping or slanting surfaces (Caziot et al., 2017; Howard & Rogers, 1995; Nakayama, 1996; Tyler, 1974).

Both Wheatstone and Julesz appreciated the benefits of their scientific work to art. Wheatstone was a friend of Talbot, the inventor of the negative/positive photographic process (see Talbot, 1844). Indeed, the first attempts at stereoscopic photography were undertaken by Talbot at Wheatstone's request (see Wade, 2021b). At that time stereoscopic photographs were taken sequentially with a single camera which was moved between exposures. The camera separations used by Talbot were too large for fusion to be possible when the positives were viewed in a stereoscope so that rivalry rather than stereoscopic depth was seen. Nonetheless, Wheatstone (1852) noted that the difficulties encountered by artists trying to represent the small differences in scenes as seen by the left and right eyes could be replaced by stereoscopic photographs: “What the hand of the artist was unable to accomplish, the chemical action of light, directed by the camera, has enabled us to effect” (1852, p. 7). The observation can be amplified when the computer is added to the camera because it was with computer-generated patterns that Julesz was able to realise Wheatstone's dream of generating stereoscopic depth without presenting clues to depth in the monocular components.

Julesz not only saw the advantages of applying his techniques to art but he also collaborated with artists. Together with Michael Noll he instigated one of the first exhibitions of computer-generated art in 1965 and exhibited some of his own works (see Noll, 2016). He interacted with Dali over several years with regard to stereoscopic paintings (Julesz, 1995), Thus, the impact of the stereoscope and stereoscopic depth perception on art has been significant and stretches back to the very origins of the enterprise. The present article is seen as trying to continue this tradition by building bridges between visual science and visual art. It also seeks to further the use of extended surfaces in depth as initiated by Wheatstone (1838) and to contrast it to the enclosed textural stereograms introduced by Julesz. To this end, a wider range of graphic textures than RDSs act as carrier patterns for stereoscopic depth, these include graphics (drawings and paintings), photographs and combinations of the two (photographics) using computer graphics (see Wade, 2021a, 2023a, 2023b, 2024). Often the stereoscopic effect is not visible initially and so some patience might be required for the depth to emerge. Julesz remarked on this and saw the virtues of slowing down the perceptual processes involved:

When these unfamiliar pictures are viewed stereoscopically, peculiar and often unexpected depth effects can be seen. In addition, the perception time of depth under such circumstances is sometimes in the order of minutes (instead of the few milliseconds required for familiar stereo images). This slowing down of the visual process facilitated the present investigation without having much effect on the stability of depth impression after depth was finally perceived. (Julesz, 1960, p. 1126)

**Figure 1.**
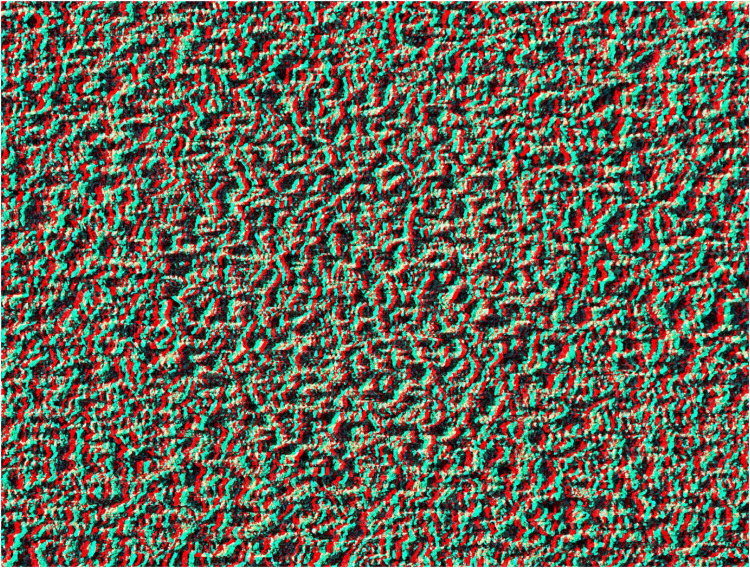
*Discovery* by Nicholas Wade. An anaglyph in which a central disc appears in depth; the carrier pattern was derived from a photograph of rain-pitted sand on a beach. With the red filter in front of the left eye (red/LE) and the cyan filter in front of the right eye (cyan/RE) the disc appears to be nearer than the background whereas with cyan/LE and red/RE the disc appears both larger and more distant. Apparent size varies with apparent distance so that when the disc appears nearer (equivalent to crossed fusion) it also appears smaller than when it appears more distant (uncrossed fusion) despite the disparity being the same.

The textured surfaces in depth are presented as anaglyphs which require red/cyan viewers to separate the half-images and expose the apparent depth. It will probably be the case that one eye/filter arrangement will produce more compelling depth than the other, which is a combination of eye dominance and differences between the filters. Unlike conventional stereoscopic photographs, reversing the filters will reverse the depths seen. The patterns can display both enclosed and extended surface depth; an example of the former is shown in Figure 1.

Figure 1, like all the figures in this article, is an anaglyph which requires red/cyan viewers to see the depth within it; the monocular members (half-images) of the stereogram are presented in colours such that the left eye (LE) component is passed by one colour filter and blocked by that in front of the right eye (RE). The first stereoscopes, invented by Wheatstone (1838, 1852), were based on mirrors, lenses and prisms. The use of colours for separating the eyes to see depth was realised by Rollmann (1853) and developed later by D’Almeida (1858) and by Ducos du Hauron (1897) who devised a method of over-printing red and blue or green designs in 1891. Thereafter, anaglyphs became increasingly popular as a means for printing and projecting stereograms. Computer software for separating the half-images has improved markedly relative to the early anaglyphs (Templin, 2016). The stereograms shown here were composed using StereoPhoto Maker software (https://stereo.jpn.org/eng/stphmkr/). The software combines the two half-images with colour separations to match those of red and cyan viewers and enables adjustments to their alignment and sizes prior to producing the final anaglyph. The textures themselves have often been manipulated extensively in Photoshop prior to their preparation for StereoPhoto Maker.

The carrier pattern in Figure 1 is a photograph of sand on a beach in which a circular area is displaced in one half-image but not in the other in order to produce the patterns seen by viewing through either the red or cyan filter alone. The monocular patterns appear to be flat but are seen in depth when the anaglyph is viewed through both filters. The conventional arrangement of filters and eyes for stereo photographs is red/LE and cyan/RE. However, this constraint is not required for the illustrations here because the stereograms have been constructed rather than derived from two photographs; either combination of eyes and filter will yield stereoscopic depth but the sign (nearer or farther) will change. As noted above, it can take some time for the depth to emerge; initially there is the impression of depth in some region and it articulates over time. There are large individual differences in the time required to see stereoscopic depth and this is particularly the case for extended surface stereoscopy (Gillam et al., 1988).

## Simple Surfaces in Depth

**Figure 2.**
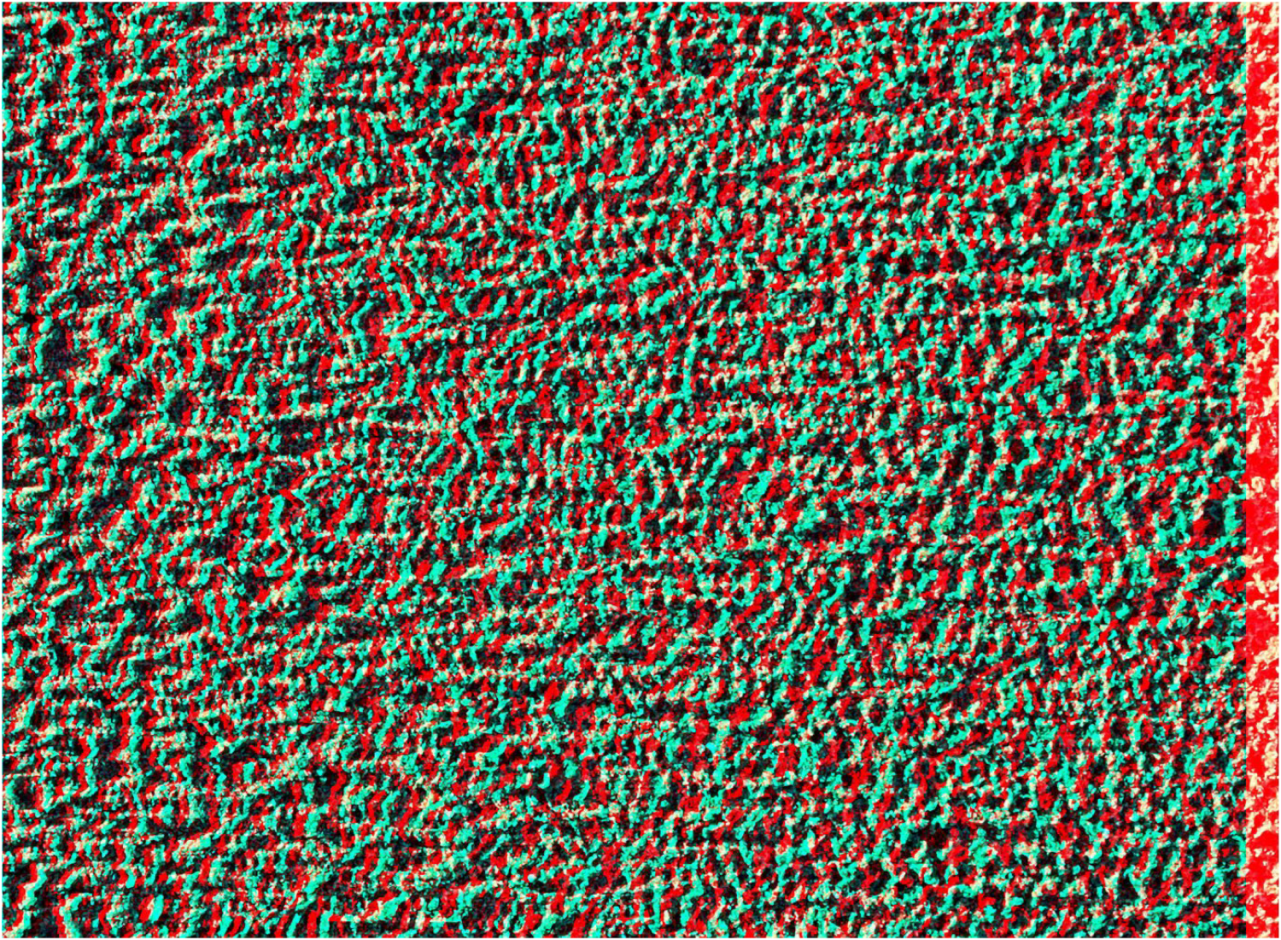
*Slanted sand* by Nicholas Wade. An anaglyph, like Figure 1, derived from a photograph of sand on a beach. Initially it appears flat but longer observation with red/LE and cyan/RE results in the right side of the background appearing both more distant and larger than the left side whereas with cyan/LE and red/RE the reverse occurs.

Modulation of the apparent depth over a surface can be achieved in several ways. The principle of rendering in depth a single spatial dimension of a stereogram was demonstrated by Wheatstone (1838). One of his stereograms was comprised of a horizontal line of dots with different separations in each eye; when viewed stereoscopically the line appeared slanted in depth (see Wade, 2025, for an example of this). That is, a single dimension (horizontal separation between the dots) determined the plane in which a line appeared in depth. This principle is extended to a surface in Figure 2 where a single dimension (width) determines the disparities which define the slant of the whole surface. It can be seen that the image visible through the cyan filter is wider than that seen through the red filter. Note that while only one dimension (width) is physically manipulated in the two half-images, two perceived dimensions change: when the right side is seen nearer its height appears vertically shorter than that on the left; conversely, when the right side appears more distant it is also seen as longer vertically. The carrier patterns for Figures 1 and 2 are derived from the same photograph of sand but the surface depth in Figure 2 takes longer to emerge than the appearance of the disc in depth in Figure 1.

**Figure 3.**
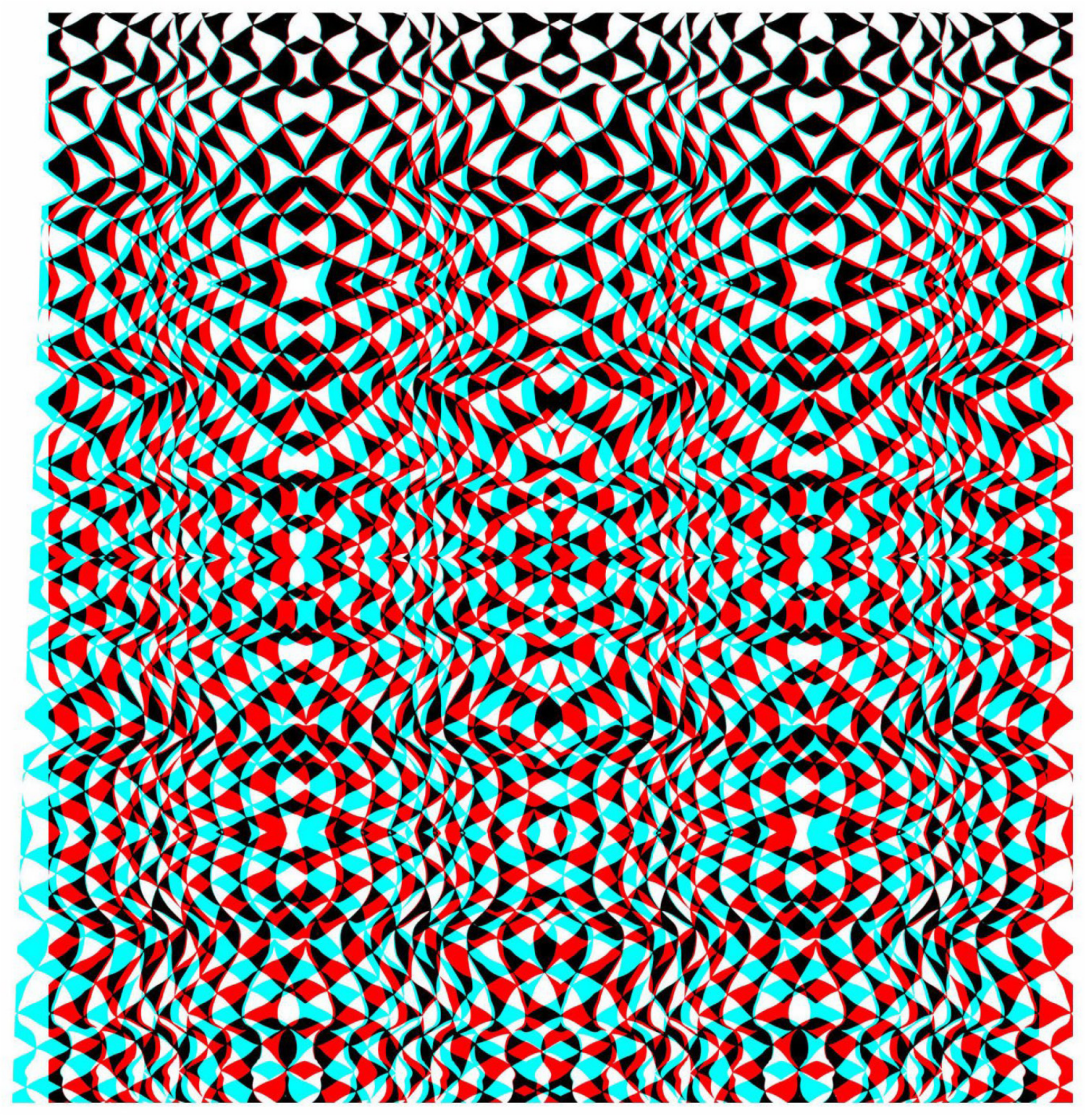
*Swing state* by Nicholas Wade. The carrier pattern is based on a graphic design which is slightly tilted from top to bottom (in opposite directions for the half-images) so that the base appears to be either closer or further away, depending on the arrangement of the red/cyan viewers.

The horizontally slanted surface stereo induced in Figure 2 is a consequence of the horizontal differences between the half-images resulting in varying degrees of disparity from left to right. Constructing shifts in the vertical dimension (as in Figure 3) involves varying the degree of horizontal disparities from top to bottom.

**Figure 4.**
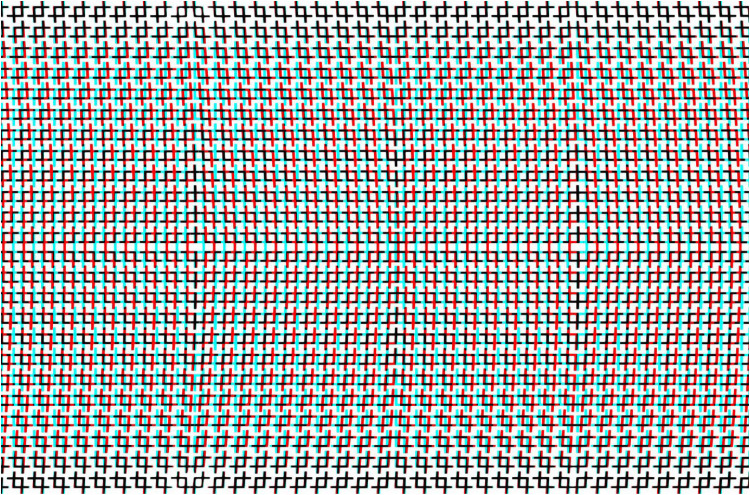
*Concave/convex* by Nicholas Wade. The carrier pattern is derived from a digitised graphical image. One half-image has been distorted and combined with an undistorted partner presented to the other eye. The anaglyph appears concave (central horizontal more distant) with red/LE and cyan/RE and convex (central horizontal diameter closer) with cyan/LE and red/RE. The apparent depth increases with longer viewing.

Curvature in depth can be introduced by combining images curved in opposite directions or by combining a curved image presented to one eye with an undistorted image in the other (Figure 4). The two half-images are in appropriate alignment and accommodation when the illusory diagonals are clearly seen.

**Figure 5.**
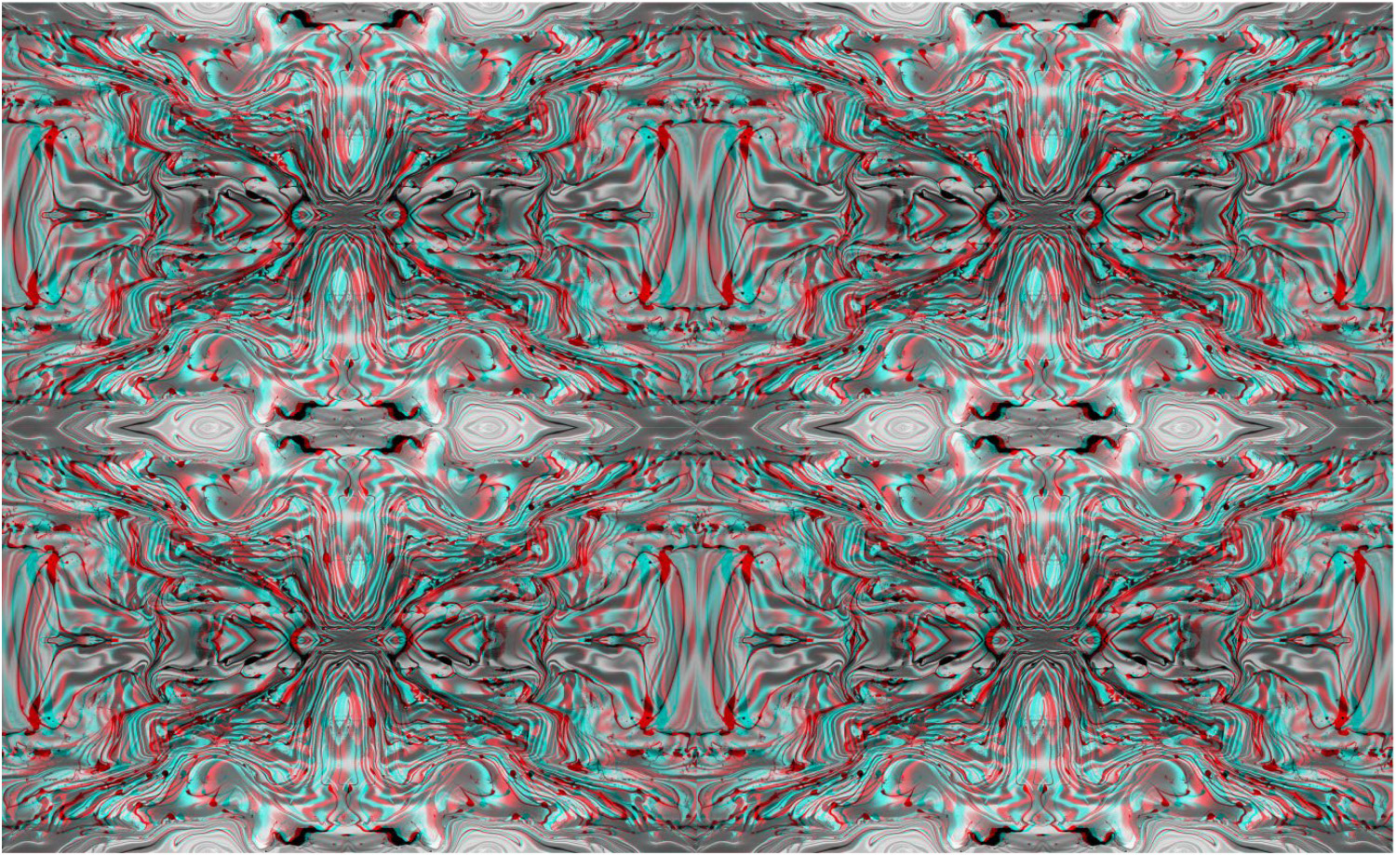
*Wave signs* by Nicholas Wade.

Surface depth modulations can incorporate concave and convex appearances within the same anaglyph, as in Figure 5. The carrier pattern is derived from a digitised image of a painting that has in turn been digitally manipulated before the half-images were made and then combined.

**Figure 6.**
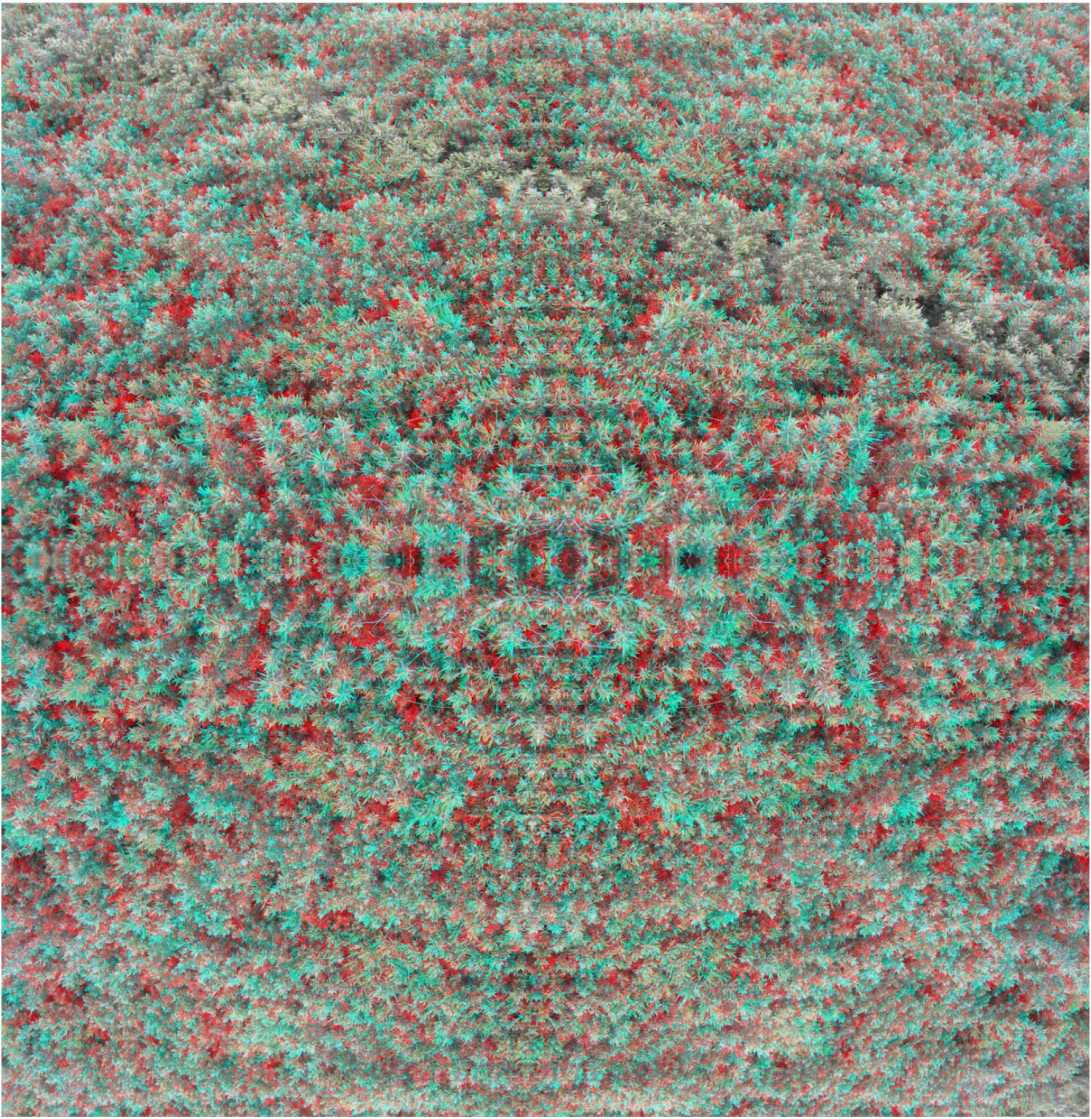
*Thorny surface* by Nicholas Wade.

Two depth manipulations can be incorporated in the same anaglyph, as in Figure 6. A pattern, derived from a photograph of thorns on a gorse bush, appears not only to be on a curved surface but this also bends at the corners. The resulting appearance is of a concave or convex surface approaching or receding at opposite corners. The curvature appears before the emergence of the corners bending.

## Complex Surfaces in Depth

**Figure 7.**
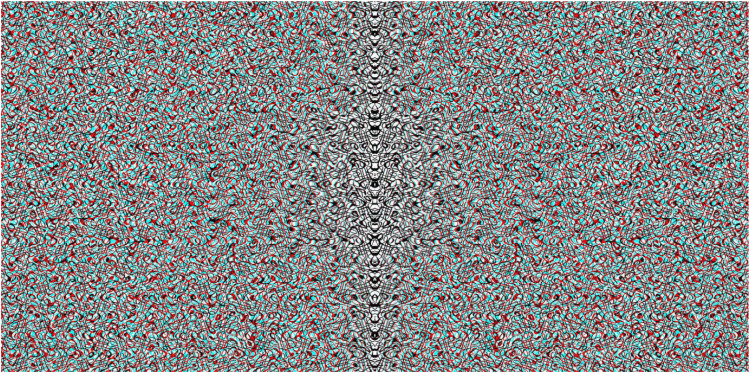
*Signs posted* by Nicholas Wade. What initially appears like a flat surface gradually changes into a slanted background with a central, horizontal band slanted in the opposite direction.

The stereoscopic surfaces that are visible in Figures 2 to 6 have been constructed from half-images that could be displayed individually. This does not apply to those that are referred to as complex stereoscopic surfaces because they are constructed from parts of, or multiple, independently derived anaglyphs. It is difficult to see how such stereo images could be constructed other than with anaglyphs. That is, anaglyphs superimpose the two monocular views whereas they are displayed separately in optical stereoscopes; the anaglyph can be likened to the internal combination of the half-images. Julesz (1971) referred to the site of the internal image as the cyclopean retina. Whereas anaglyphs can be cropped and combined, these processes cannot be applied to the cyclopean image, nor to the half-images presented in optical stereoscopes. The complex surface anaglyphs could be ‘deconstructed’ to produce separate half-images for viewing in an optical stereoscope but this would not be straightforward. An example of a complex stereoscopic surface is shown in Figure 7 which is based on a single anaglyph one side of which was reversed and then combined with the original. Each half has a central, horizontal strip slanted in the opposite direction to its surround; the depths in the left and right halves are also opposite so that it gives the impression of a single strip running through the slanted surround like a signpost.

**Figure 8.**
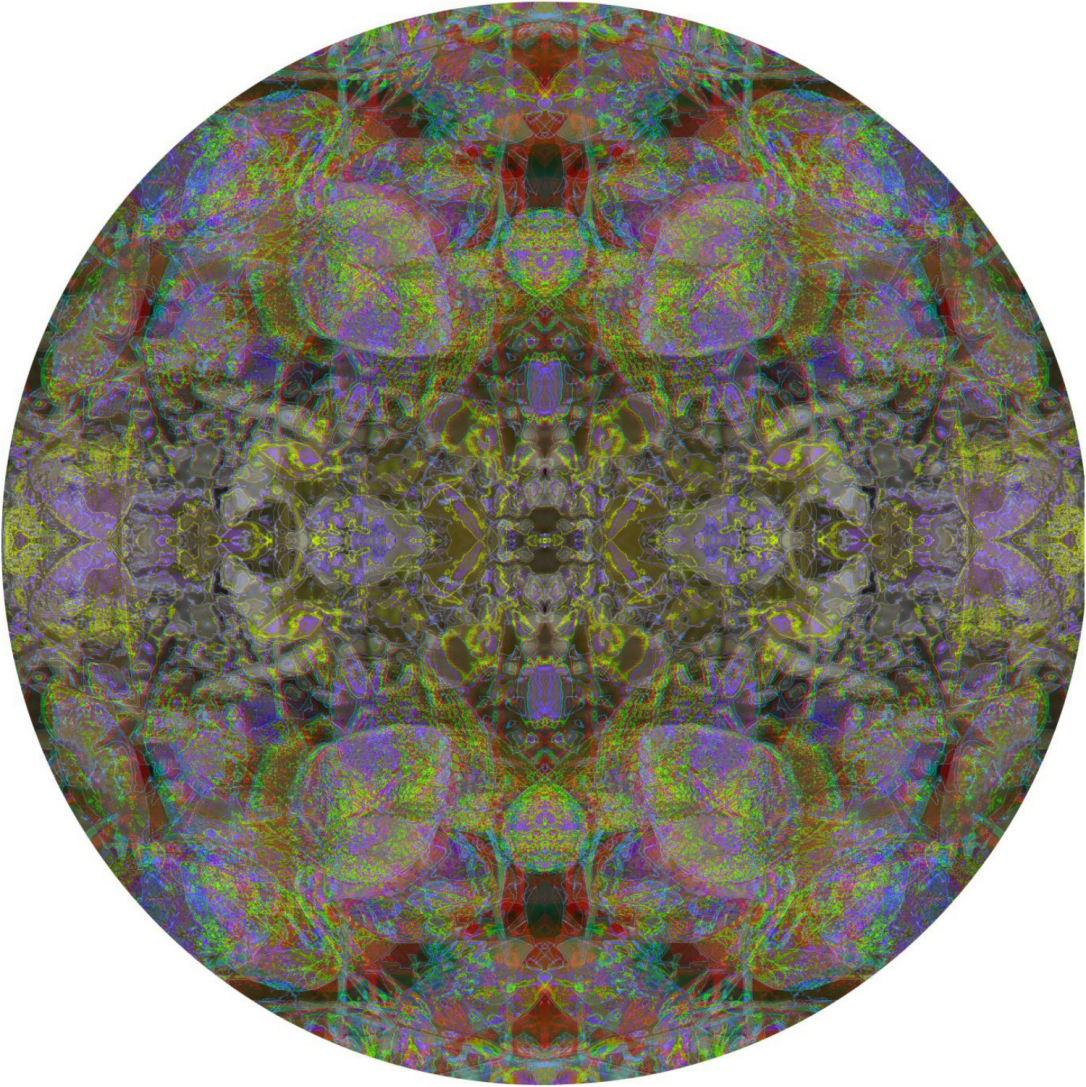
*Separation* by Nicholas Wade. The left and right halves of the anaglyph appear to cleave away from one another along a vertical axis with one appearing concave and the other convex.

Figure 8 is based on a photograph of leaves on a blueberry bush that initially appears flat but with longer observation the left and right halves separate in depth with one side convex and the other concave. The image was made by producing an anaglyph with the disparities to yield a uniformly curved surface; it was then bisected vertically and one half was rotated about a vertical axis and rejoined to its unrotated half. This results in the apparent curvature in opposite directions, as if the two halves are shearing away from one another.

**Figure 9.**
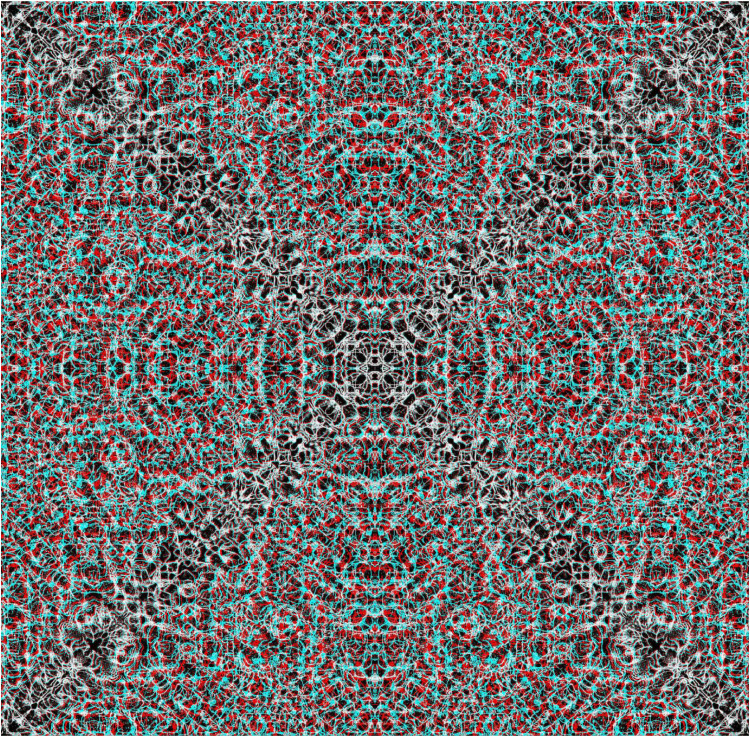
*Propeller* by Nicholas Wade.

The depth separation in Figure 8 is along a vertical axis whereas this can occur in both vertical and horizontal axes. Figure 9 was constructed from a single anaglyph with an apparent rotation in depth along the diagonal axes. The single anaglyph was rotated by 90 degrees three times and the components were combined to form the completed anaglyph. That is, Figure 9 is composed of four anaglyphs, each one at right angles to its neighbours.

**Figure 10.**
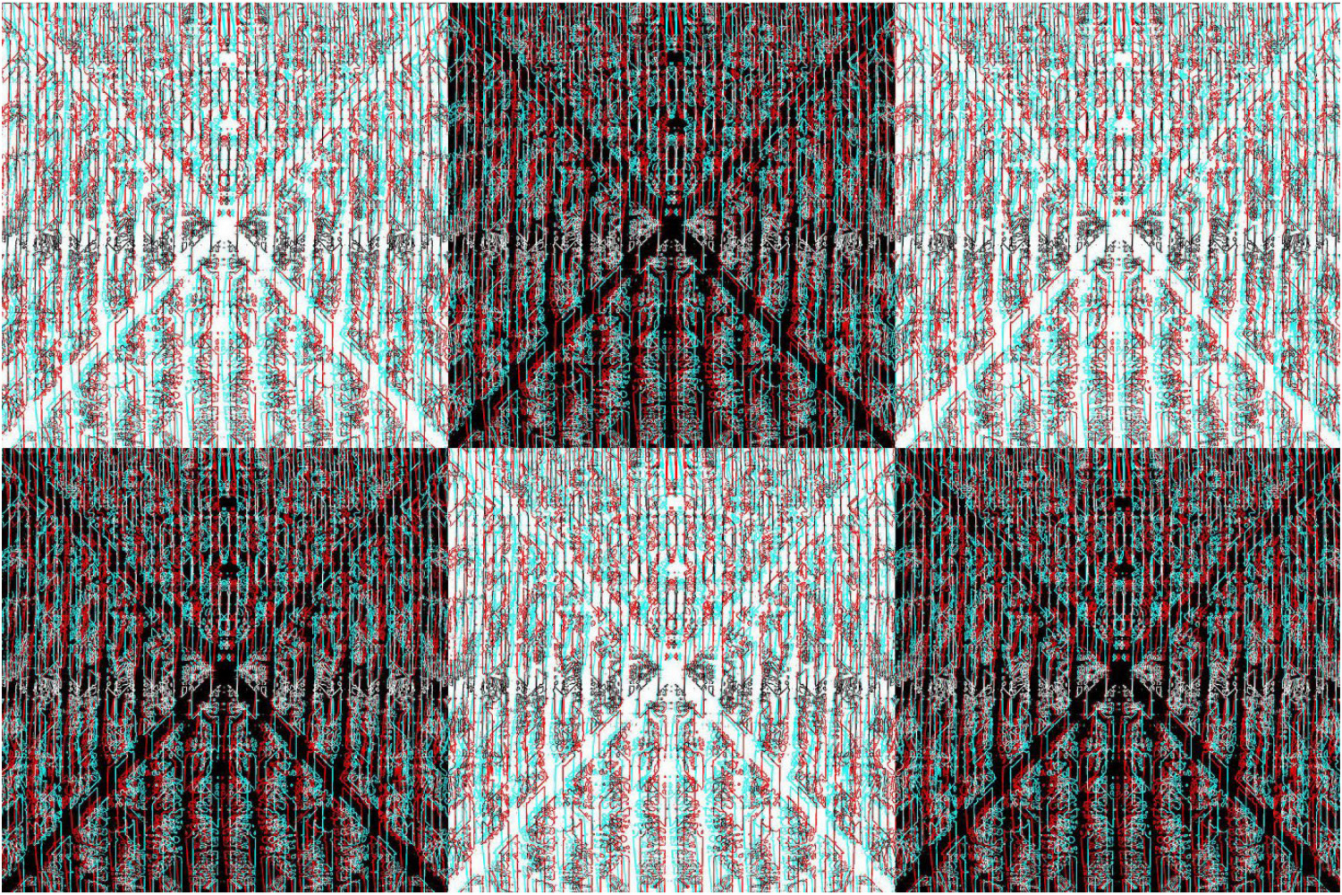
*X-waves* by Nicholas Wade.

Much more complex stereo surfaces can be constructed from combinations of anaglyphs that themselves have several distortions, as shown in Figure 10. The carrier pattern is derived from a painting and a single anaglyph with a surface wave was constructed. Both the positive and negative of the anaglyph were triplicated and combined in the manner shown in Figure 10 so that the diagonals were aligned and the surface depth was unified throughout the stereoscopic image.

**Figure 11.**
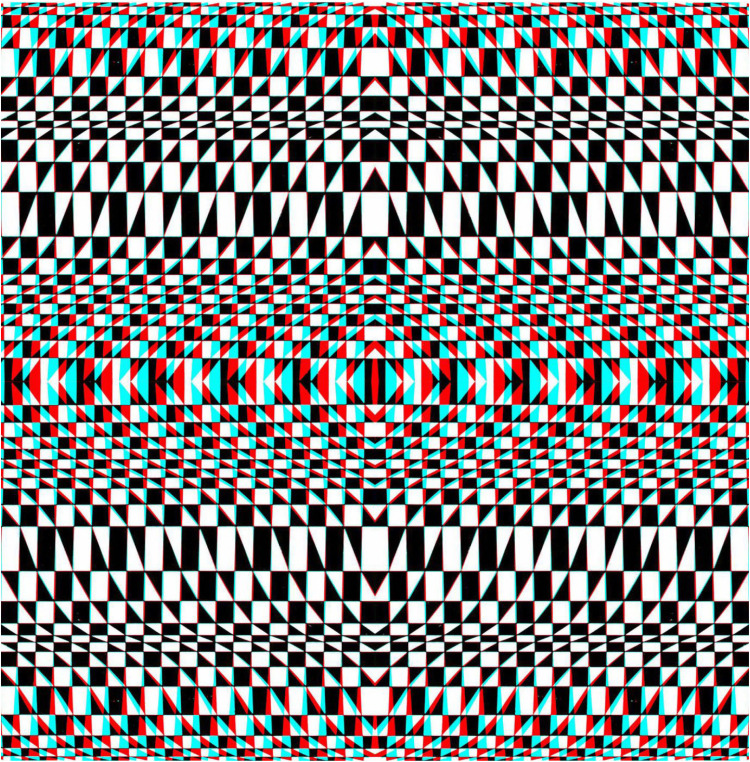
*Twisted triangles* by Nicholas Wade.

As with simple surface stereoscopy, the apparent depths can be reversed in composite anaglyphs. An example is shown in Figure 11 in which the undulations in depth are reversed on the left and right sides. The image is a combination of four anaglyphs each of which consists of triangles and rectangles of different sizes. Despite all the lines being straight there is the impression of curvature in the continuities of the hypotenuses of the triangles.

**Figure 12.**
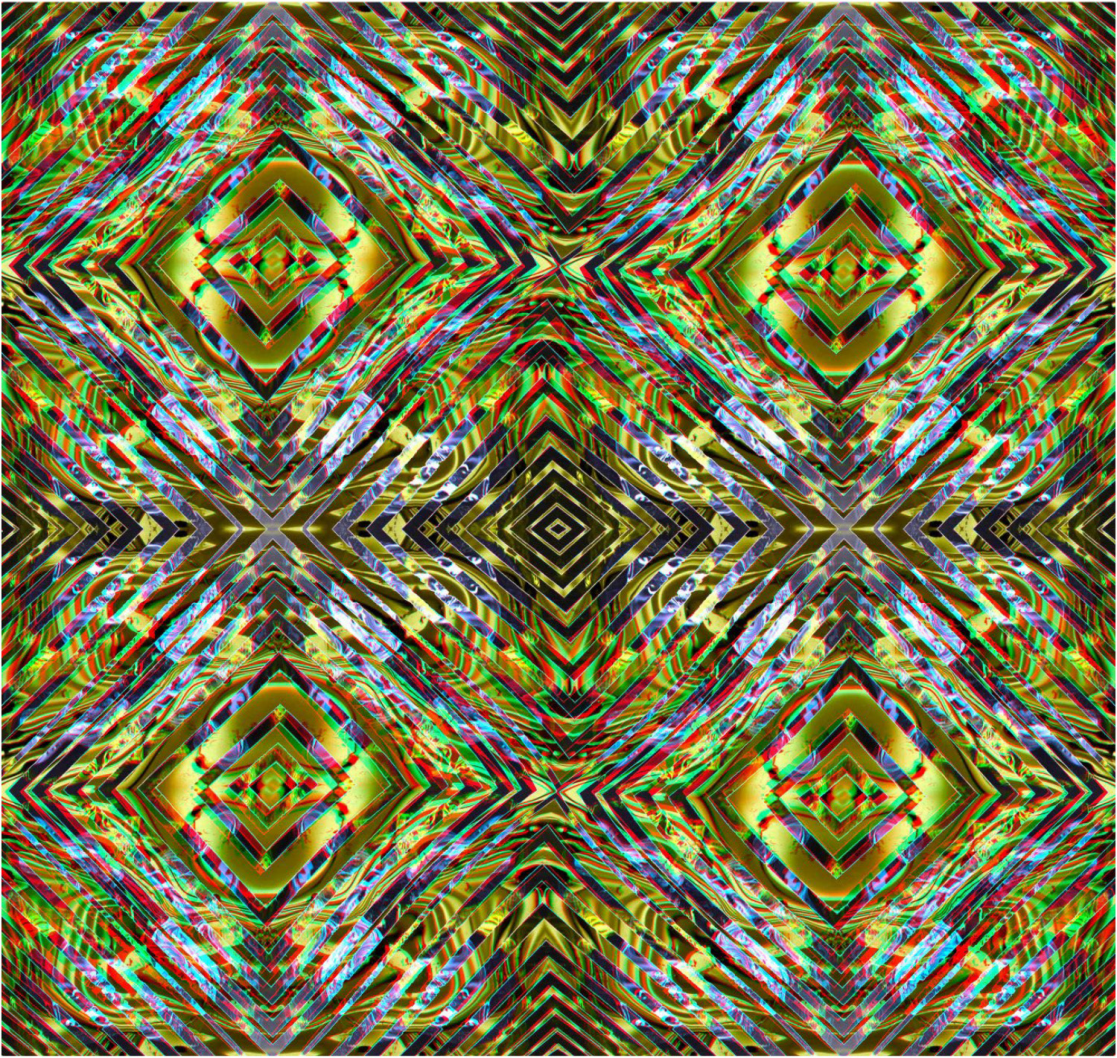
*Topology* by Nicholas Wade.

Surface stereoscopy can be variable within a complex anaglyph so that the surface modulations can be difficult to describe but are readily visible. Different deformations in depth were created in the independent parts that are combined in Figure 12. The stereoscopic surface seems to rise and fall while coursing its way through the anaglyph, rather like an aerial view of a landscape. The hills turn into valleys when the filter/eye arrangement is reversed and then all appears flat through either the filter alone.

## Surface Stereo Combined With Inclusions in Depth

Figure 1 presented a simple stereogram with enclosed surface depth. Extended surfaces in depth can be more complex as can the enclosures within them. As with the anaglyphs in section 3, such modulations could not be produced for conventional stereoscopes because there are no equivalent half-images that could be constructed.

**Figure 13.**
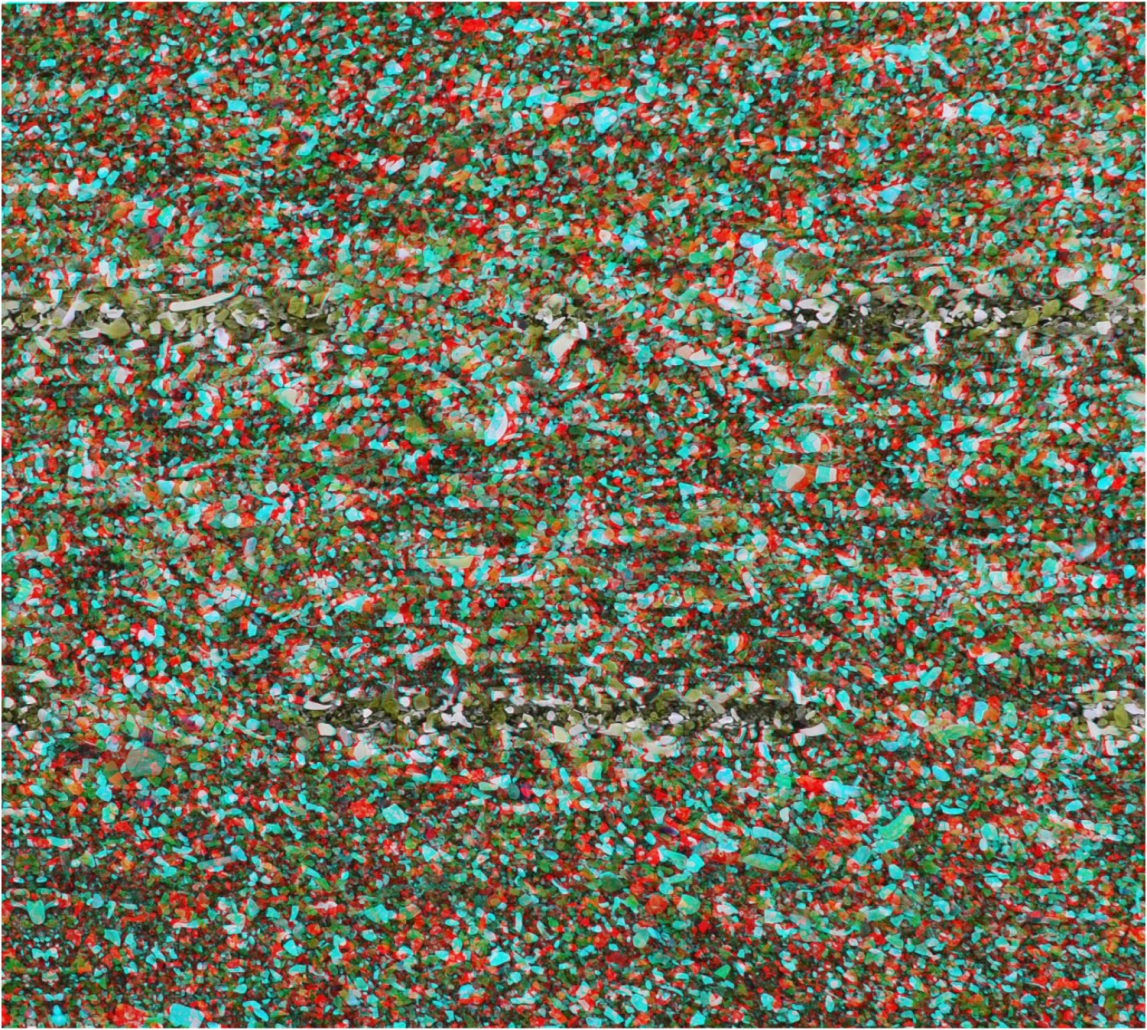
*Kanisza's curved triangle* by Nicholas Wade. Viewing the anaglyph with red/LE and cyan/RE results in the appearance of a convex surface with enclosed incomplete discs defining an illusory triangle which is also convex but apparently more distant; the reverse occurs for the extended and enclosed surfaces with cyan/LE and red/RE.

The carrier pattern for Figure 13 is derived from a photograph of broken shells on a beach. The extended surface appears concave or convex, depending on the arrangement of the red and cyan filters. Within the design three incomplete discs, with their missing sectors aligned, form a Kanisza triangle; it can be seen with the same curvature as the surround but the incomplete circles defining it are seen at a different depth.

**Figure 14.**
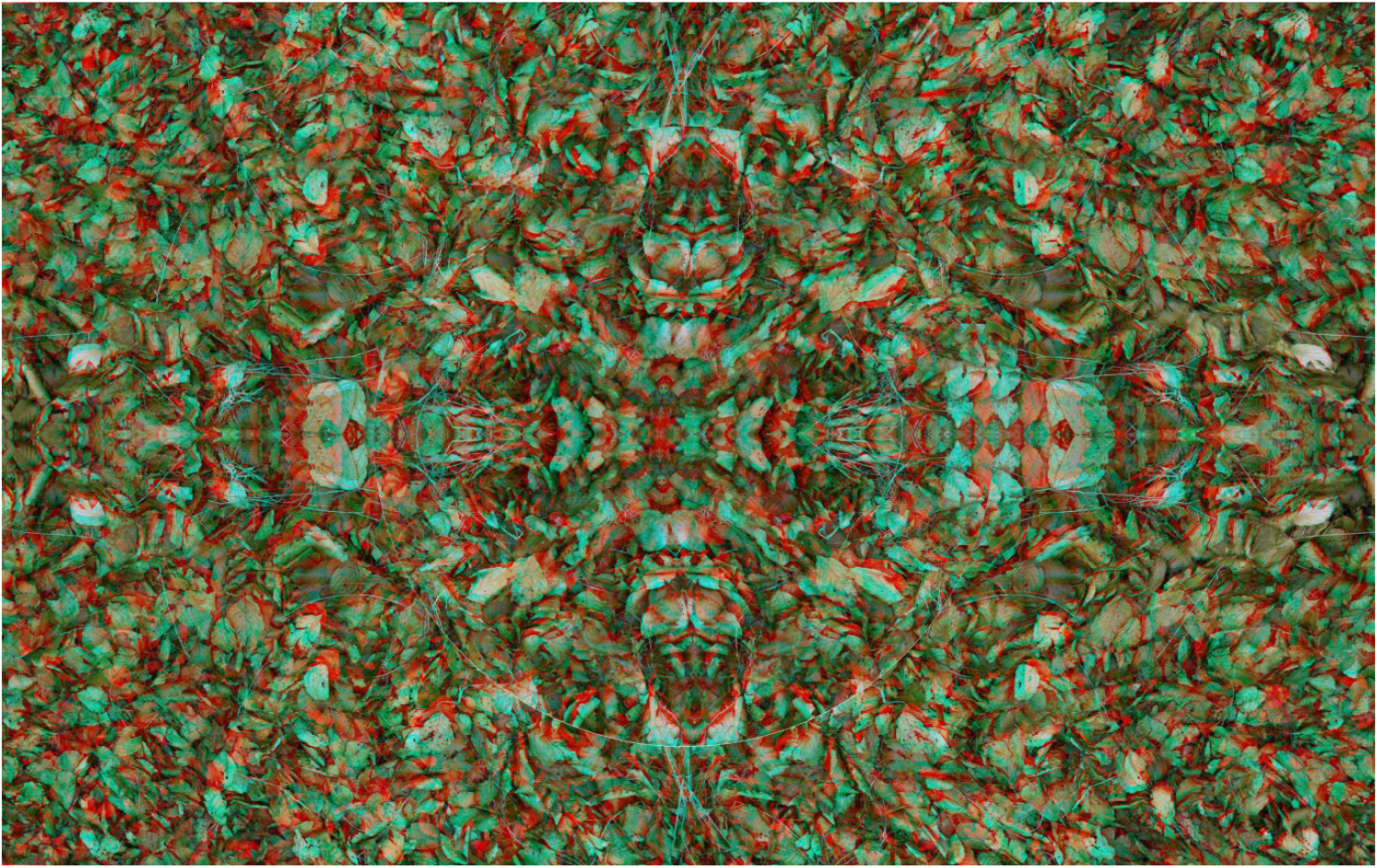
*Releaf* by Nicholas Wade. An enclosed central oval has the opposite curvature in depth to the extended surround. The whole surface is slanted from left to right; with red/LE and cyan/RE the right side appears closer with the reverse for cyan/LE and red/RE.

The enclosed curvature in Figure 13 is in the same direction as that enclosing it whereas the relationship in Figure 14 is reversed. The carrier pattern is derived from a photograph of autumn leaves on the ground and the undulation in depth that is visible in the extended region is reversed in the central circular area. In addition to the waves of depth from top to bottom, the whole surface is slanted from left to right.

**Figure 15.**
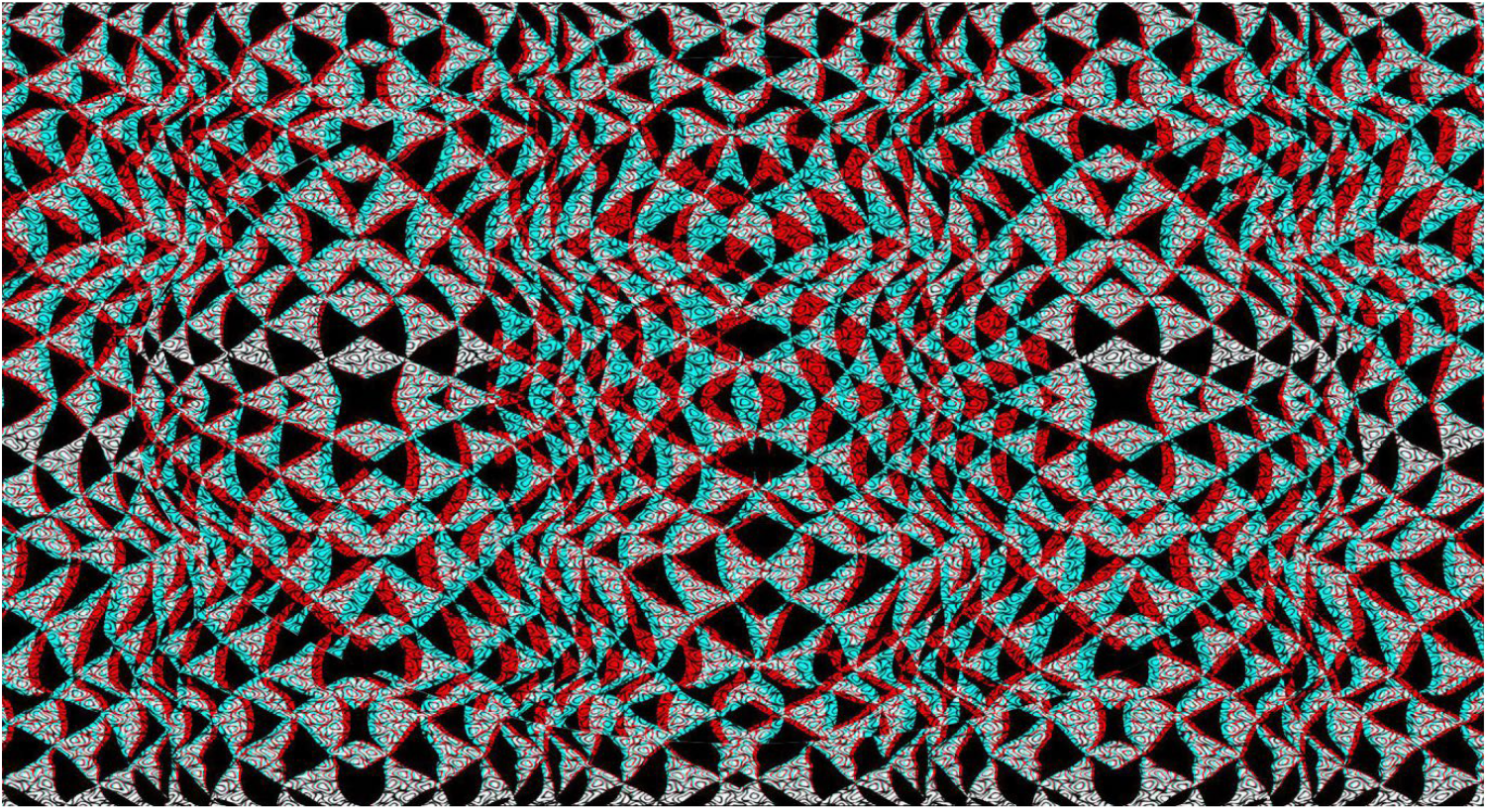
*Eye seas* by Nicholas Wade.

Both the extended and enclosed surfaces can be more intricate than is the case for Figures 13 and 14. That is, rather than varying, say, the curvature from top to bottom over the whole surface a region within it can have the opposite curvature in depth. In Figure 15 an enclosed ellipse has within it a circular area but their apparent depths differ; the curvature is seen to change in the same directions but at different depths. The shape of the extended surface is similarly modulated from top to bottom but in the opposite direction.

**Figure 16.**
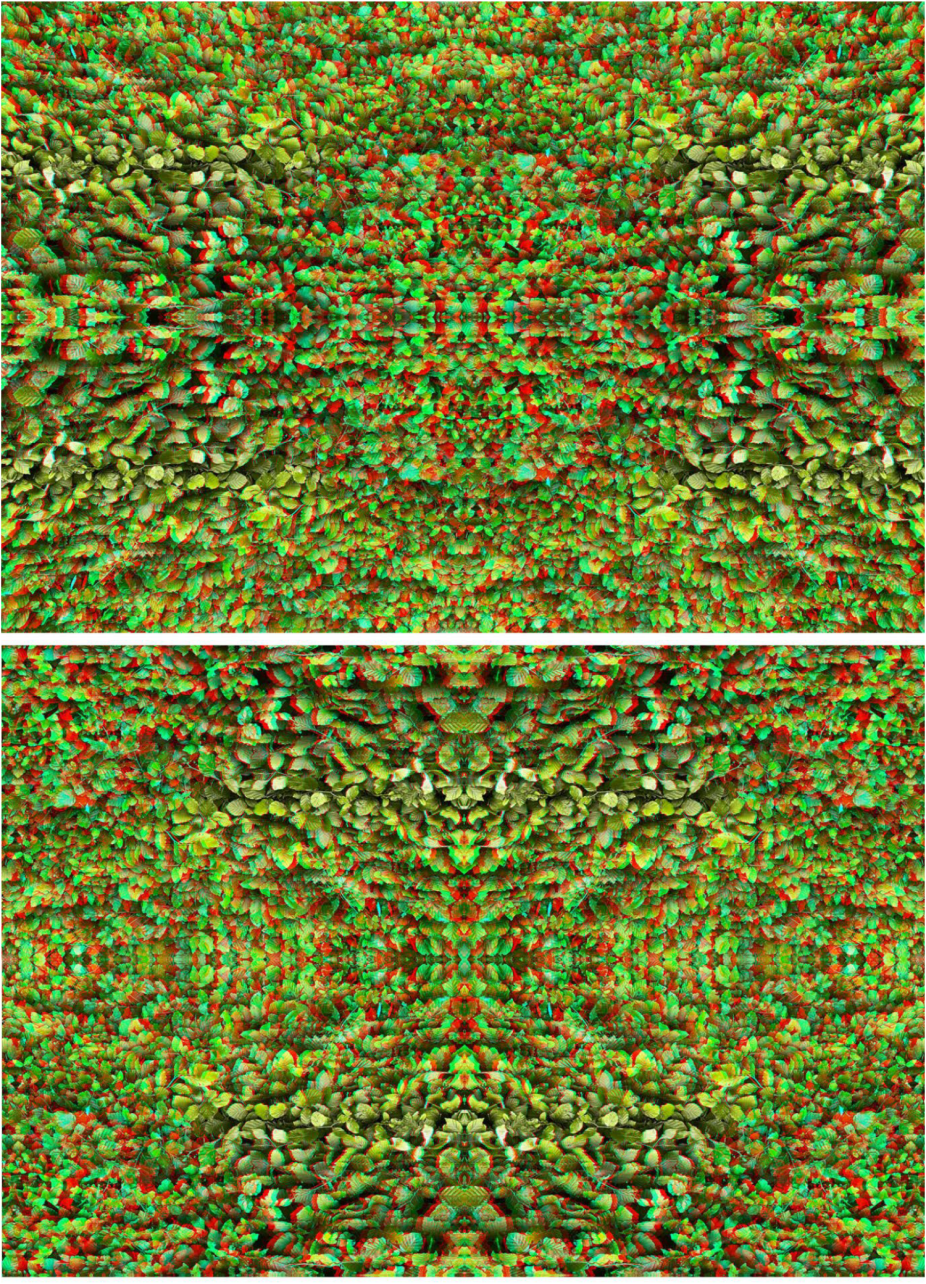
Upper, *Beech discs* and lower, *Beech quadrants* both by Nicholas Wade.

It is also possible to produce an anaglyph comprised of extended and enclosed depths, dissect it and then reassemble the parts. Figure 16 provides an example of this by displaying the anaglyph, derived from a photograph of beech leaves, in its original and transformed states. The initial anaglyph consisted of extended curvature and enclosed concentric circles at different apparent depths. It was then quartered and the quadrants were reassembled so that the central points of the original are now the four corners of the anaglyph.

**Figure 17.**
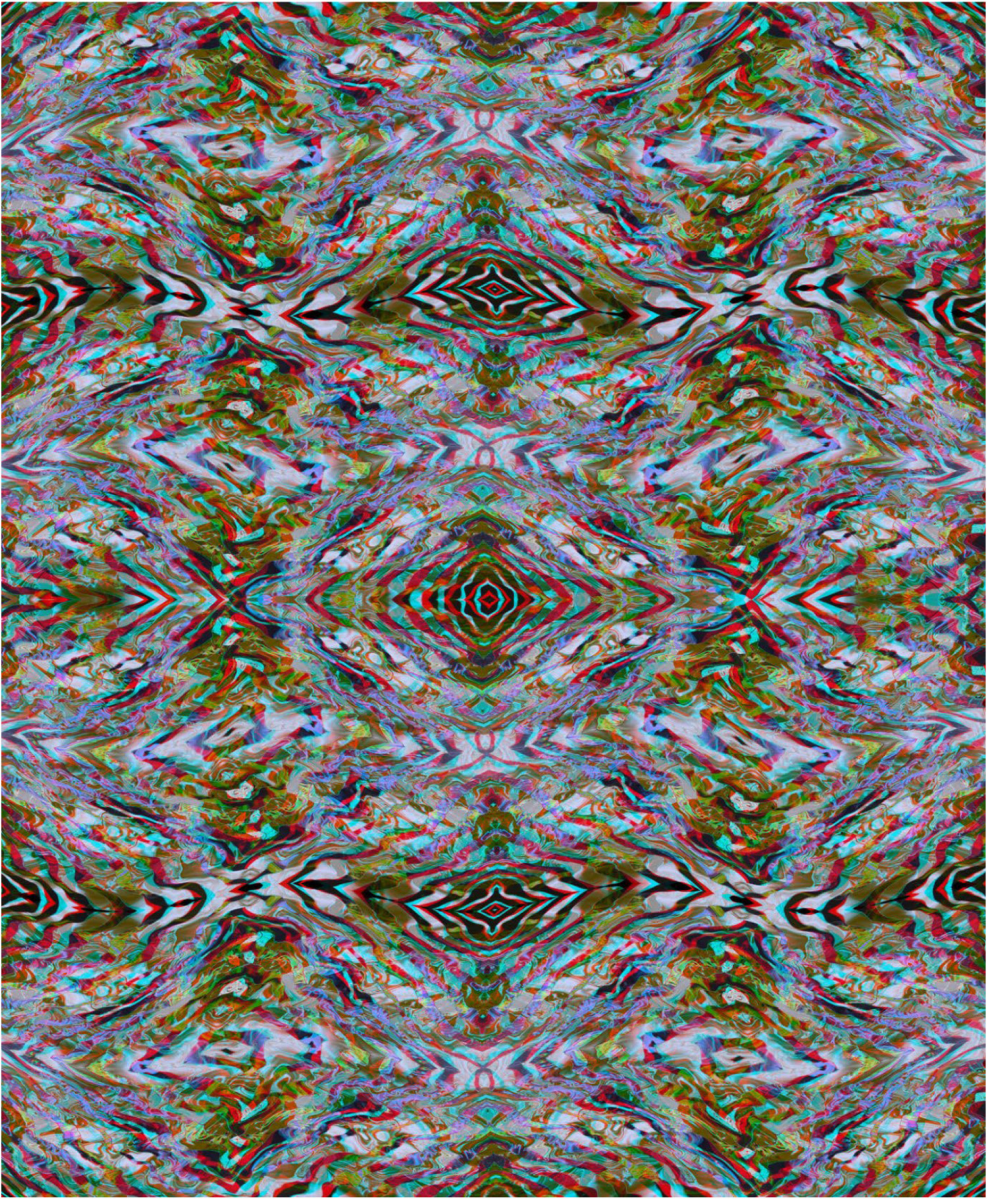
*Wandering waves* by Nicholas Wade.

In addition to dissecting and recombining anaglyphs, they can be produced and then distorted; Figure 17 is an elaborate example of this. The initial design was a combination of two paintings, one geometrical and the other more free-flowing. The regular geometrical features were distorted before an initial anaglyph was formed; it was multiplied six times to make the final combination which was then distorted itself. Prior to the final distortion the transition between the depths on the left and right sides of the anaglyph were readily apparent but that is not so afterwards.

## Constructed and Conventional Stereoscopy Combined

**Figure 18.**
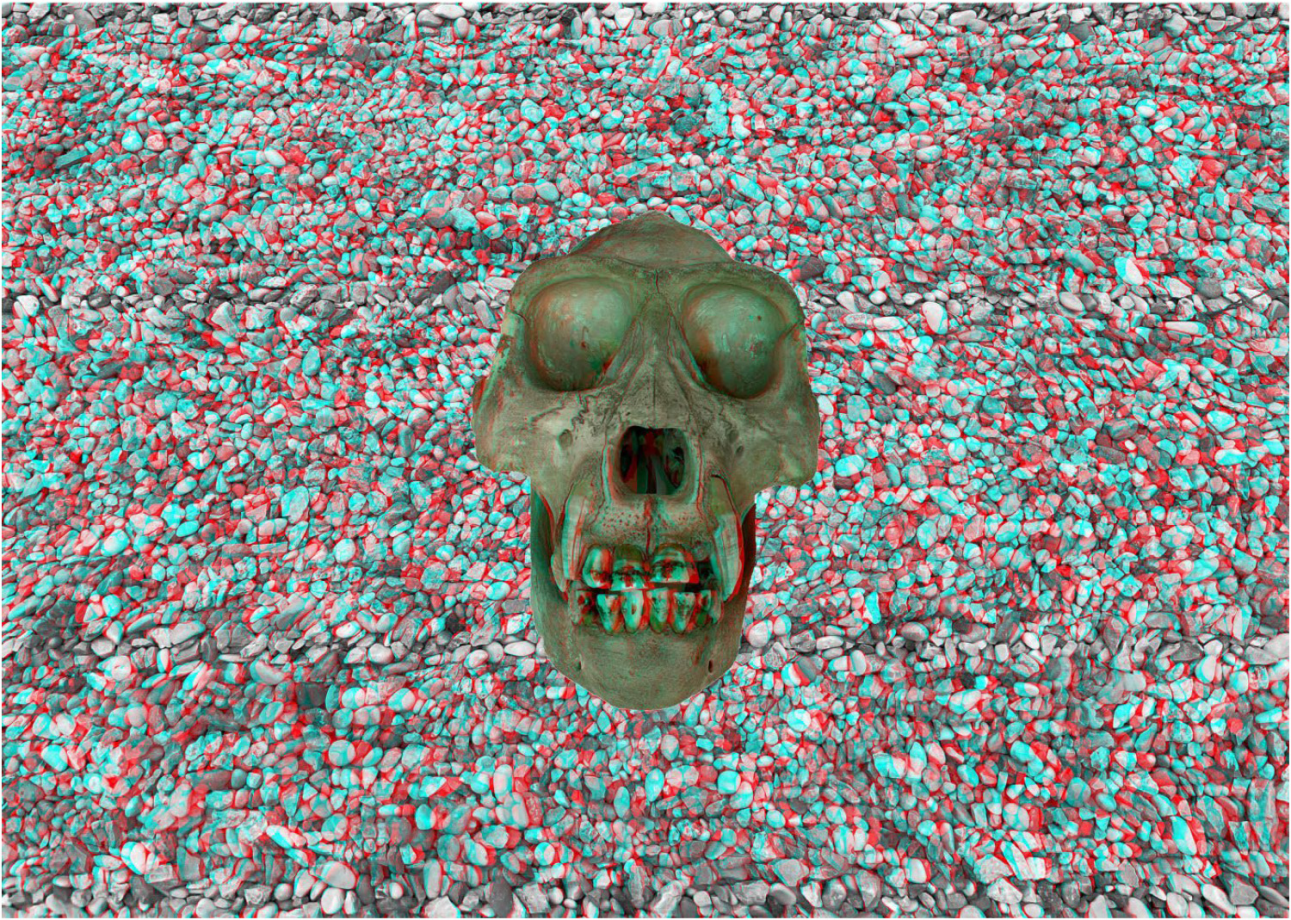
*Primate skull* by Nicholas Wade.

Conventional stereograms refer to those that are taken photographically, either by a single-lensed camera moved between two exposures or using a twin-lensed stereo camera. The distinction between constructed and conventional stereograms is that the former display reversed depth with reversal of the viewing eyes (or red/cyan filters) whereas the latter do not. Figure 18 provides an example of this distinction. The stereoscopic photograph of a primate's skull is placed within a textured surface stereogram. When viewed with red/LE and cyan/RE the depth in the orbits and nasal region of the skull are seen within a stony background that has a wavy surface depth. When the red/cyan viewers are reversed the signs of the depths in the background reverse but the skull does not reverse in apparent depth. In the former case it appears as though the solid skull lies in a trough running through the stones.

**Figure 19.**
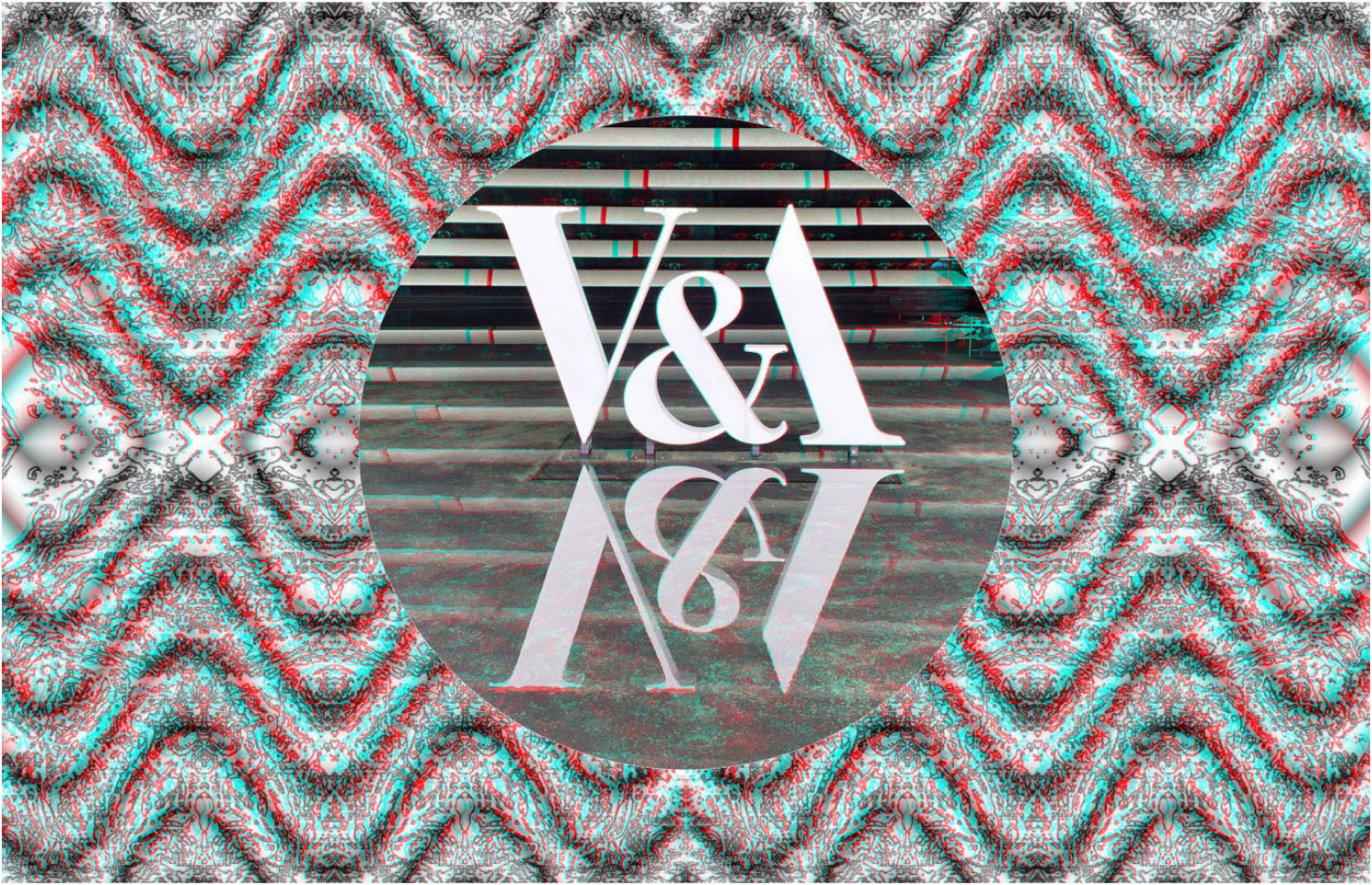
*Virtual & Actual* by Nicholas Wade.

The conventional stereoscopic image in Figure 18 contains all the disparities within the subject matter – the skull; the background is formed from a photograph of stones that have been manipulated to create the appearance of curved depth from top to bottom. Figure 19 contains stereoscopic depth within the background of the conventional, central stereoscopic image. The sign for the Victoria & Albert museum in Dundee stands in a shallow pool of water so that its reflection is mirrored, as are some of the horizontal structures of the building itself. The constructed surface background is both graphically and stereoscopically wavy and it relates to the watery surround of the museum.

**Figure 20.**
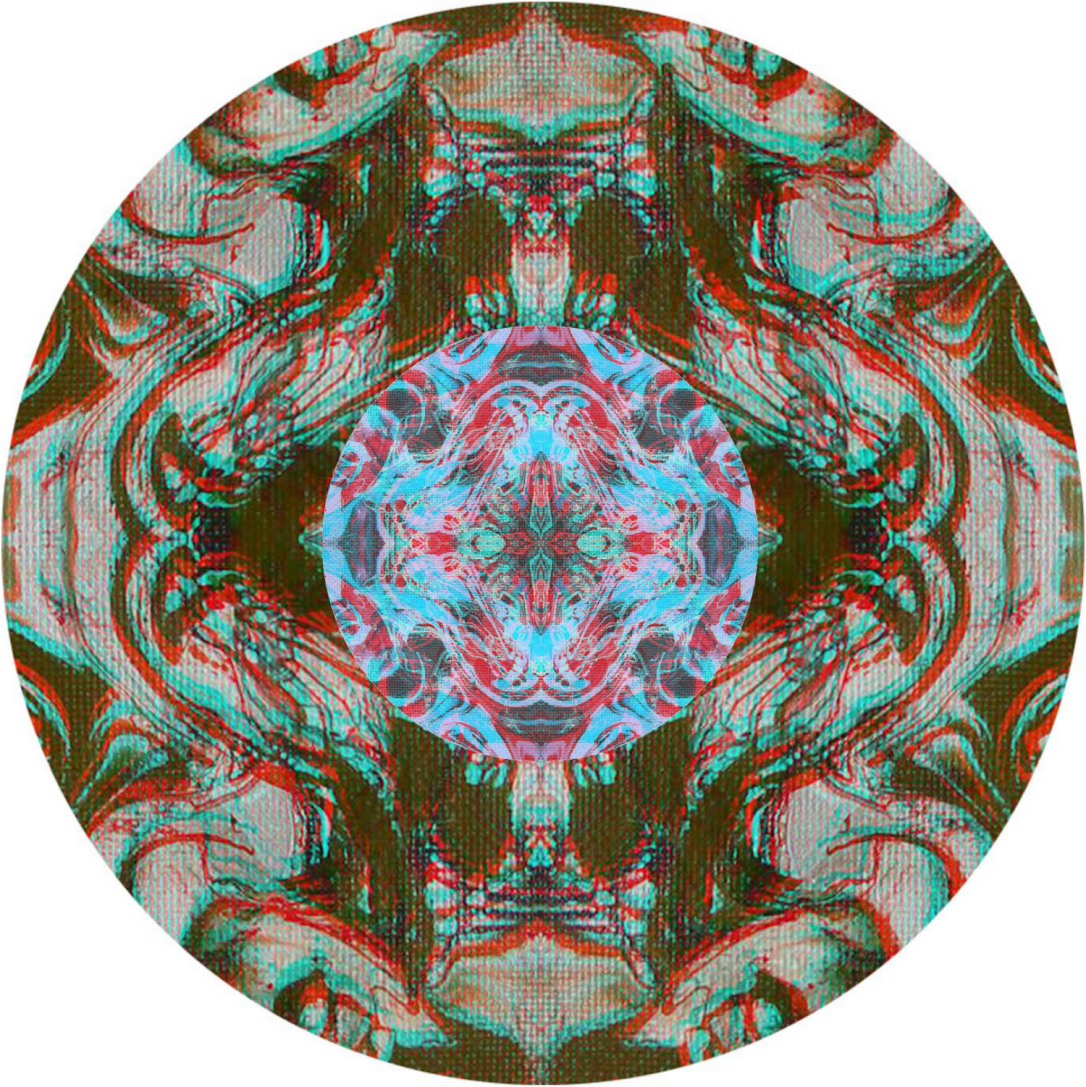
*Contrasting curves* by Nicholas Wade.

In addition to combining conventional stereoscopic images within textured backgrounds, rivalling images can also be introduced, as in Figure 20. All the components of the illustration have the same source – a flow painting on a textured board. The annulus appears folded in depth with either a saddle (red/LE and cyan/RE) or a hollow (cyan/LE and red/RE) running diagonally through it from bottom left to top right. In the centre is the same base pattern, but smaller with the two components at right angles as well as being the negative of the surround. Thus, the same pattern has been used to create the extended surround in-depth and the enclosed centre in rivalry.

**Figure 21.**
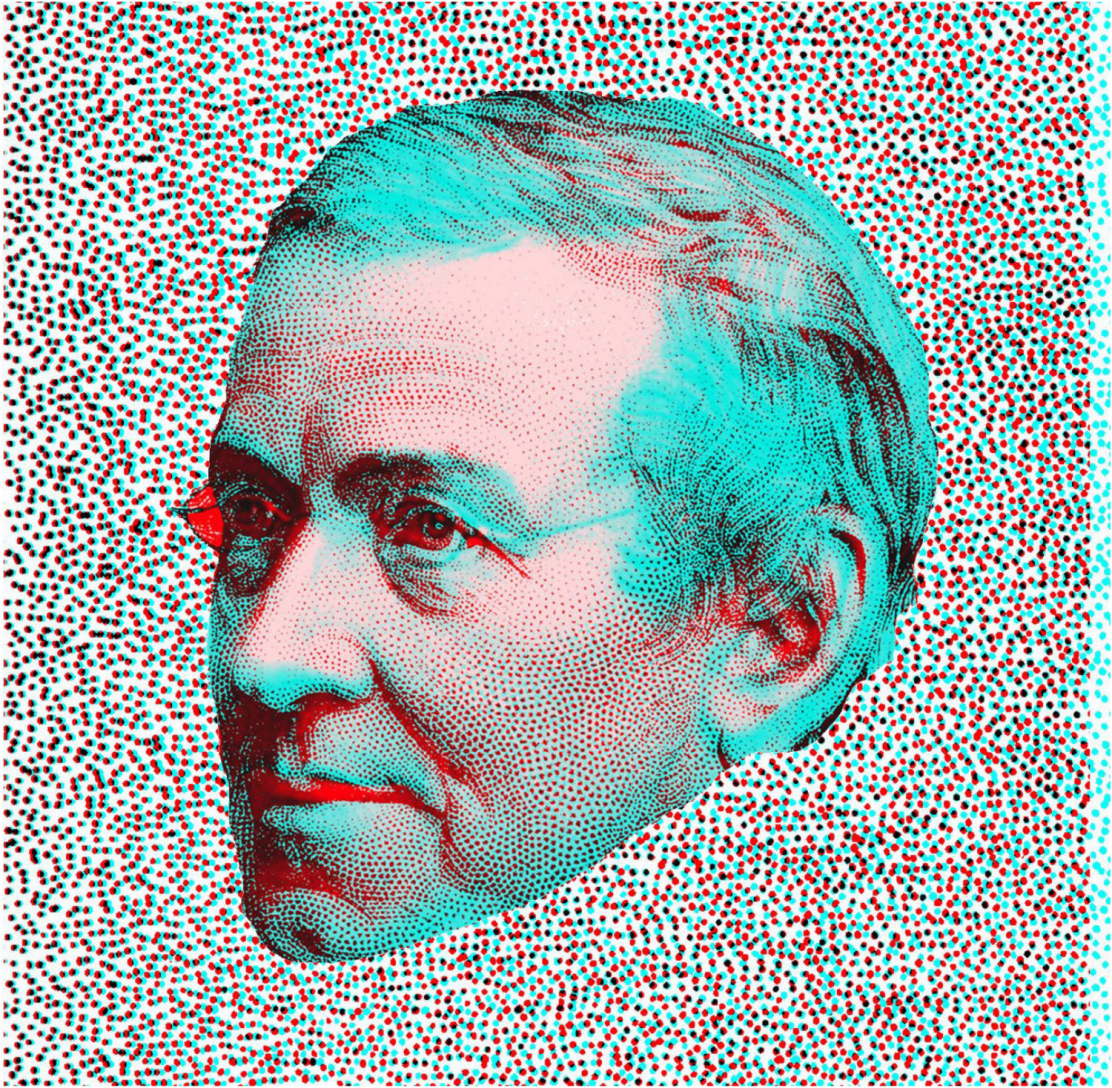
*Younger and older Wheatstone in depth* by Nicholas Wade. The texture of the surface is comprised of hand-drawn dots varying in separation in the two eyes. With red/LE and cyan/RE the right hand side appears closer than the left, and this reverses with cyan/LE and red/RE. A younger Wheatstone can be seen through the red filter and an older portrait through the cyan filter.

The final two figures present rivalling portraits of the pioneers of stereoscopy who introduced the possibilities of extended and enclosed surfaces in depth. First, Charles Wheatstone (Figure 21) is shown as a younger and older man in central rivalry against an extended background of dots; they are an elaboration of the row of dots in depth he presented in his memoir describing the invention of the stereoscope (Wheatstone, 1838). The two portraits can be seen by viewing through each colour filter in turn: the younger Wheatstone can be seen through the red filter and the older one through the cyan. When viewing the stereogram binocularly the younger and older versions rival with one another.

**Figure 22.**
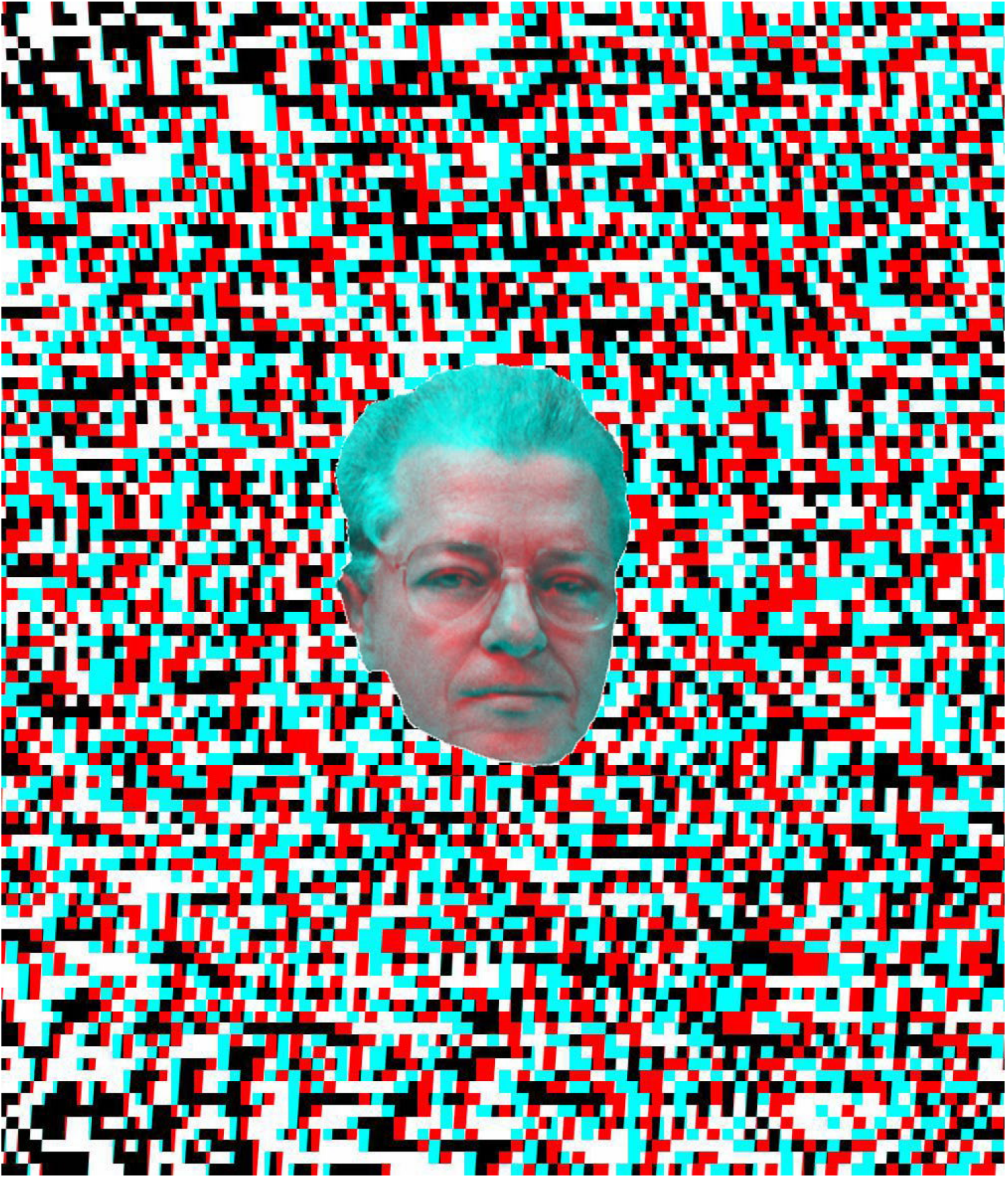
*Random-dot stereoscopist* by Nicholas Wade.

Béla Julesz is similarly shown as a younger and older man in rivalry at the centre of Figure 22. His double portrait is placed in a central stereoscopic square within a random dot pattern the surround of which appears to be both curved and slanted.

## Discussion

The enclosed textured stereograms introduced by Julesz (1960) produced stereoscopic depth without monocular recognition. In contrast, extended textures are a fundamental feature of our natural environment and are a constant aspect of our binocular vision. Can it be said that the extended surface stereograms, a precursor of which was presented by Wheatstone (1838) and many examples of which are illustrated in this article, reflect a more natural manifestation of stereoscopic depth without monocular recognition? Firstly, the function of stereoscopic vision is to detect and detach objects from the surfaces on which they lie, in addition to determining surface variations to guide body motion through the space. Secondly, the surroundings of the object detected are not uniform textures but have gradients. Indeed, texture gradients play a prominent role in Gibson's (1950) theory of spatial vision. Extended surface stereoscopy can be considered as adding gradients in one or more spatial dimensions. Thus, surfaces can appear slanting or curved in stereoscopic space, as can the enclosed surfaces within them. The distinction between enclosed and extended stereograms is important. In the case of enclosed stereograms the region surrounding the stereoscopic surface is in retinal correspondence whereas this is not the case for extended surface stereograms. For the latter, disparities vary over a surface, sometimes gradually and at others abruptly. This is likely to be a reason for the longer times required for the apparent depths to emerge.

Experimental manipulations of enclosed surface stereoscopy have added enormously to our understanding of stereoscopic depth perception in flat displays. Whether that can be matched by extended surface stereoscopy remains to be seen. However, modulating extended surfaces as well as enclosures within them does enhance the perceptual intrigue they can induce.

## Conclusion

Julesz stated that the enclosed surface stereoscopic effects produced by RDSs do not correspond to any naturally occurring scenes. This caveat does not apply in the same way to extended surface stereoscopy, which is a constant feature of binocular vision in the natural environment. Binocular fixation on any object will result in disparities generated by surrounding objects and surfaces. However, the constructed stereograms displayed in this article are presented on a plane surface and so in this sense they, too “never occur in real-life situations”. This is a characteristic common to all stereograms, no matter how they are produced. Indeed it is a feature of all the pictorial images we observe either with one or two eyes. Nonetheless, extended surface stereoscopy offers many opportunities for extending stereoscopic art.

## References

[bibr1-20416695251349685] Caziot B. Bachus B. T. Lin E. (2017). Early dynamics of stereoscopic surface slant perception. Journal of Vision, 17, 4. 10.1167/17.14.4 PMC5713489 29196763

[bibr2-20416695251349685] D’Almeida J.-C. (1858). Nouvel appareil stéréoscopique. Comptes Rendus, 47, 61–63.

[bibr3-20416695251349685] Ducos du Hauron A. (1897). La triplice photographique des couleures et l'imprimerie: systéme de photochromographie Louis Ducos du Hauron. Gauthier-Villars.

[bibr4-20416695251349685] Gibson J. J. (1950). Perception of the visual world. Houghton Mifflin.

[bibr5-20416695251349685] Gillam B. Chambers D. Russo T. (1988). Postfusional latency in stereoscopic slant perception and the primitives of stereopsis. Journal of Experimental Psychology: Human Perception and Performance, 14, 163–175. 10.1037/0096-1523.14.2.163 2967874

[bibr6-20416695251349685] Howard I. P. Rogers B. J. (1995). Binocular vision and stereopsis. Oxford University Press.

[bibr7-20416695251349685] Julesz B. (1960). Binocular depth perception of computer-generated patterns. The Bell System Technical Journal, 39, 1125–1162. 10.1002/j.1538-7305.1960.tb03954.x

[bibr8-20416695251349685] Julesz B. (1971). Foundations of cyclopean perception. Chicago University Press.

[bibr9-20416695251349685] Julesz B. (1995). Dialogues on perception. MIT Press.

[bibr10-20416695251349685] Nakayama K. (1996). Binocular surface perception. Proceedings of the National Academy of Sciences USA, 93, 634–639. 10.1073/pnas.93.2.634 PMC40103 8570607

[bibr11-20416695251349685] Noll A. M. (2016). The Howard Wise gallery show of computer-generated pictures (1965): A 50th-anniversary memoir. Leonardo, 49, 232–239. 10.1162/LEON_a_01158

[bibr12-20416695251349685] Rollmann W. (1853). Zwei neue stereoskopische methoden. Annalen der Physik und Chemie, 166, 186–187. 10.1002/andp.18531660914

[bibr13-20416695251349685] Talbot H. F. (1844). The pencil of nature. Longman, Brown, Green, & Longmans.

[bibr14-20416695251349685] Templin K. (2016). Stereo and anaglyph images. In Luo M. R. (Ed.), Encyclopedia of color science and technology (pp. 1176–1178). Springer.

[bibr15-20416695251349685] Tyler C. M. (1974). Depth perception in disparity gratings. Nature, 251, 140–142. 10.1038/251140a0 4420707

[bibr16-20416695251349685] Wade N. J. (2021a). On stereoscopic art. i-Perception, 12, 1–17. 10.1177/20416695211007146 PMC8165537 34104379

[bibr17-20416695251349685] Wade N. J. (2021b). On the art of binocular rivalry. i-Perception, 12, 1–23. 10.1177/20416695211053877 PMC8649479 34888026

[bibr18-20416695251349685] Wade N. (2023a). Vision and art with two eyes. Springer.

[bibr19-20416695251349685] Wade N. J. (2023b). Revealing the concealed: Alternatives to random-dots for stereograms. Vision, 7, 78. 10.3390/vision7040078 38133481 PMC10747603

[bibr20-20416695251349685] Wade N. J. (2024). The art of binocular (a)symmetries. Bi-Symmetrics: The Journal of Symmetry & Asymmetry. 1, 9–25. https://img1.wsimg.com/blobby/go/881b5455-24b3-4d60-ba89-78898f85ef86/downloads/Wade.pdf?ver=1714214792032

[bibr21-20416695251349685] Wade N. J. (2025). Stereoscopic depth without monocular recognition. i-Perception, 16, 1–16. 10.1177/20416695251329309 PMC12035379 40297835

[bibr22-20416695251349685] Wheatstone C. (1838). Contributions to the physiology of vision—part the first. On some remarkable, and hitherto unobserved, phenomena of binocular vision. Philosophical Transactions of the Royal Society, 128, 371–394. 10.1098/rstl.1838.0019

[bibr23-20416695251349685] Wheatstone C. (1852). Contributions to the physiology of vision – part the second. On some remarkable, and hitherto unobserved, phenomena of binocular vision. Philosophical Transactions of the Royal Society, 142, 1–17. 10.1098/rstl.1852.0001

